# Correlation Between Clinical Factors and Pregnancy Outcome Following Repeat Cerclage: A Retrospective Analysis of a Chinese Population

**DOI:** 10.3389/fmed.2022.846755

**Published:** 2022-04-04

**Authors:** Benshuo Cai, Yajun Xia, Xinni Na

**Affiliations:** Department of Obstetrics and Gynecology, Shengjing Hospital of China Medical University, Shenyang, China

**Keywords:** repeat cerclage, gestational age, prolapsed membranes, pregnancy outcome, retrospective analysis

## Abstract

**Background:**

The role of repeat cerclage (RC) among patients with prolapsed membranes remains controversial. We aimed to investigate the effectiveness of RC and assess the correlation between clinical factors and pregnancy outcome following RC.

**Methods:**

The clinical data of patients who underwent RC for prolapsed membranes after prior cerclage were retrospectively investigated. The clinical characteristics of patients were compared between singleton and twin pregnancies. The clinical characteristics of singleton pregnancies were compared between the gestational age (GA) at delivery <28 weeks' and ≥28 weeks' groups. Receiver operating characteristic (ROC) curve analysis was performed to determine predictive factors. Singleton patients were divided into two groups according to GA at RC as follows: GA <22.3 weeks and GA ≥22.3 weeks. Pregnancy outcomes were compared between groups.

**Results:**

The mean GA at delivery of singleton pregnancies was significantly higher than that of twin pregnancies. The mean latency between RC and delivery of singleton pregnancies was significantly longer than their twin counterparts. There were significant differences in the pregnancy outcomes between the GA <22.3 weeks group and GA ≥22.3 weeks group. Kaplan–Meier survival curves showed a lower incidence of neonatal death in the GA ≥22.3 weeks group compared with that in the GA <22.3 weeks group.

**Conclusions:**

RC may be an effective method to prolong the duration of pregnancy among patients with singleton pregnancy. However, the selection of RC for patients with twin pregnancies remains controversial. GA at RC appears to be fair for predicting pregnancy outcomes following RC.

## Introduction

Cervical cerclage is a surgical intervention involving the placement of a stitch around the uterine cervix, with the aim of preventing cervical effacement and dilatation. However, women with cervical insufficiency remain at a high risk of second-trimester loss and preterm birth despite transvaginal cerclage suture (1, 2). Cervical cerclage indicated following clinical finding of prolapsing fetal membranes at the external cervical orifice is called an emergency cerclage. Cerclage has been reported to prolong gestational age (GA) and improve the pregnancy outcome; however, it is also known to increase the risk of preterm premature rupture of membranes, chorioamnionitis, and other complications (3–13).

The pregnancy outcome following emergency cerclage is affected by various clinical factors, such as nulliparity, *in vitro* fertilization, prolapsed membranes, cervical dilation, positive vaginal culture, GA at operation, infection, type of suture, and adjunctive pessary therapy (14). Prolapse of membranes following cervical cerclage is usually considered as cerclage failure. Repeat cerclage (RC), also called reinforcing cerclage, is a special kind of emergency cerclage that is considered a remedy for cerclage failure. Because of the high risk for rupture of membranes and delivery before neonatal viability after the procedure, only cases with prolapsed membranes after prior cerclage are chosen to undergo RC. Reports on the epidemiology of RC are limited, and the results obtained are conflicting in nature (15–21). Moreover, the clinical factors affecting pregnancy outcomes following RC have hardly ever been reported.

The aim of this study was to investigate the effectiveness of RC and assess the correlation between clinical factors and pregnancy outcome following RC in a Chinese population.

## Materials and Methods

### Patients and Study Design

In this retrospective cohort study, the clinical data of patients who underwent RC at the Shengjing Hospital of the China Medical University between January 2015 and December 2020 were evaluated. The study protocol was approved by the Institutional Review Board of the Shengjing Hospital of China Medical University, Shenyang, Liaoning Province, China (Approval number: 2020PS819K). Written informed consent was obtained from the patients for their participation in the study and the publication of this report.

The study inclusion criteria were as follows: GA between 16 and 28 weeks; cervical dilation ≥1 cm with prolapsed membranes, intact membranes, no uterine contraction, no vaginal bleeding, and no fetal abnormalities. Women with maternal complications such as infections or autoimmune diseases, chorioamnionitis, and incomplete clinical information were excluded.

The RC procedure (McDonald-type) (22) was performed under combined spinal epidural anesthesia using 5 mm Mersilene® tape (RS 22) or 10^#^ non-absorbable surgical sutures. The previous cerclage knot was removed. All cerclage procedures were performed by experienced senior obstetricians. After the procedure, patients were advised bed rest and administered antibiotics and atosiban for at least 48 h; simultaneously, progesterone was continuously administered vaginally after discharge until delivery.

The clinical characteristics of patients were compared between singleton and twin pregnancies. Furthermore, the clinical characteristics of singleton pregnancies were compared between GA at delivery <28 weeks and ≥28 weeks. In addition, singleton patients were divided into two groups according to GA at RC as follows: GA <22.3 weeks and GA ≥22.3 weeks, and pregnancy outcomes were compared between these groups.

### Statistical Analysis

The data were compared between the two groups using the analysis of variance or Mann–Whitney U test. The difference in percentages between the groups was compared using Fisher's exact probability test. The receiver operater characteristic (ROC) curves were analyzed to assess the discriminative ability of GA at RC. Survival curves were compared using Kaplan–Meier analysis and the log-rank test. A *p* < 0.05 was considered statistically significant. All statistical analyses were performed using SPSS 23.0 statistical software (IBM Corp., Armonk, NY, USA).

## Results

Repeat cerclage procedures were performed successfully in 30 patients. There were no cases of membrane rupture or immediate pregnancy loss during the procedures. All the patients were Chinese. Eight patients had a twin pregnancy, and one patient experienced intrauterine fetal death of one twin. Table 1 shows the comparison of clinical characteristics between singleton and twin pregnancies. The mean GA at delivery of singleton pregnancies was 28.7 weeks, with a neonatal survival rate of 68.2% (15/22). This was significantly higher than that of twin pregnancies. In addition, the mean latency between RC and delivery of singleton pregnancies was 3 weeks longer than that in their twin counterparts. Among the 30 pregnancies, 11 ended with immediate neonatal death due to extreme prematurity. Nine patients delivered vaginally following removal of the cerclage knot, and the remaining 10 patients underwent cesarean section due to scarred uterus, fetal malposition, oligohydramnios, or fetal distress.

**Table 1:** Comparison of clinical characteristics between patients with singleton and twin pregnancy.

**Variables**	**Groups**		***p***
	**Singleton**	**Twins**	
	**(*n* = 22)**	**(*n* = 8)**	
Age (years)	33.7 ± 3.2	33.3 ± 4.9	0.760
*In vitro* fertilization (%)	3/22 (13.6)	6/8 (75)	0.003
Gravida	3.3 ± 1.1	2.4 ± 1.3	0.066
Indication of prior cerclage	–	–	
History (%)	13/22 (59)	4/8 (50)	0.698
Ultrasound/Physical examination (%)	9/22 (41)	4/8 (50)	0.698
GA at prior cerclage (week)	16.2 ± 2.5	17.7 ± 4.1	0.280
GA at repeat cerclage (week)	23.8 ± 3.0	23.4 ± 2.9	0.741
Type of suture	–	–	
Mersilene® tape (RS 22) (%)	6/22 (27.3)	2/8 (25)	1.000
10^#^ non-absorbable surgical suture (%)	16/22 (72.7)	6/8 (75)	1.000
GA at delivery (weeks)	28.7 ± 4.5	24.8 ± 2.5	0.027
Cervical dilation (cm)	2.3 ± 1.0	1.5 ± 0.9	0.066
Cervical length (cm)	1.0 ± 1.0	0.9 ± 1.0	0.805
Ureaplasma urealyticum infection (%)	6/22 (27.3)	1/8 (12.5)	0.638
Gestational diabetes mellitus (%)	8/22 (36.4)	2/8 (25)	0.682
Latency between repeat cerclage and delivery (day)	34.9 ± 27.2	10.8 ± 8.0	0.017
Neonatal birth weight (g)	1,184 ± 677	709 ± 309	0.026
Live birth rate (%)	15/22 (68.2)	9/15 (60)	0.730
Neonatal survival rate (%)	15/22 (68.2)	5/15 (33.3)	0.050
Neonatal hospitalization rate (%)	13/15 (86.7)	7/9 (77.8)	0.615
Apgar score ≤ 7 at 5 min (%)	2/15 (13.3)	4/9 (44.4)	0.150
NICU admission (%)	11/15 (73.3)	7/9 (77.8)	1.000
Chorioamnionitis (%)	3/22 (13.6)	3/8 (37.5)	0.300
Puerperal infection (%)	4/22 (18.2)	3/8 (37.5)	0.345

*Values are presented as n, mean ± standard deviation, or n/N (%)*.

*GA, gestational age; NICU, neonatal intensive care unit*.

**Figure 1:**
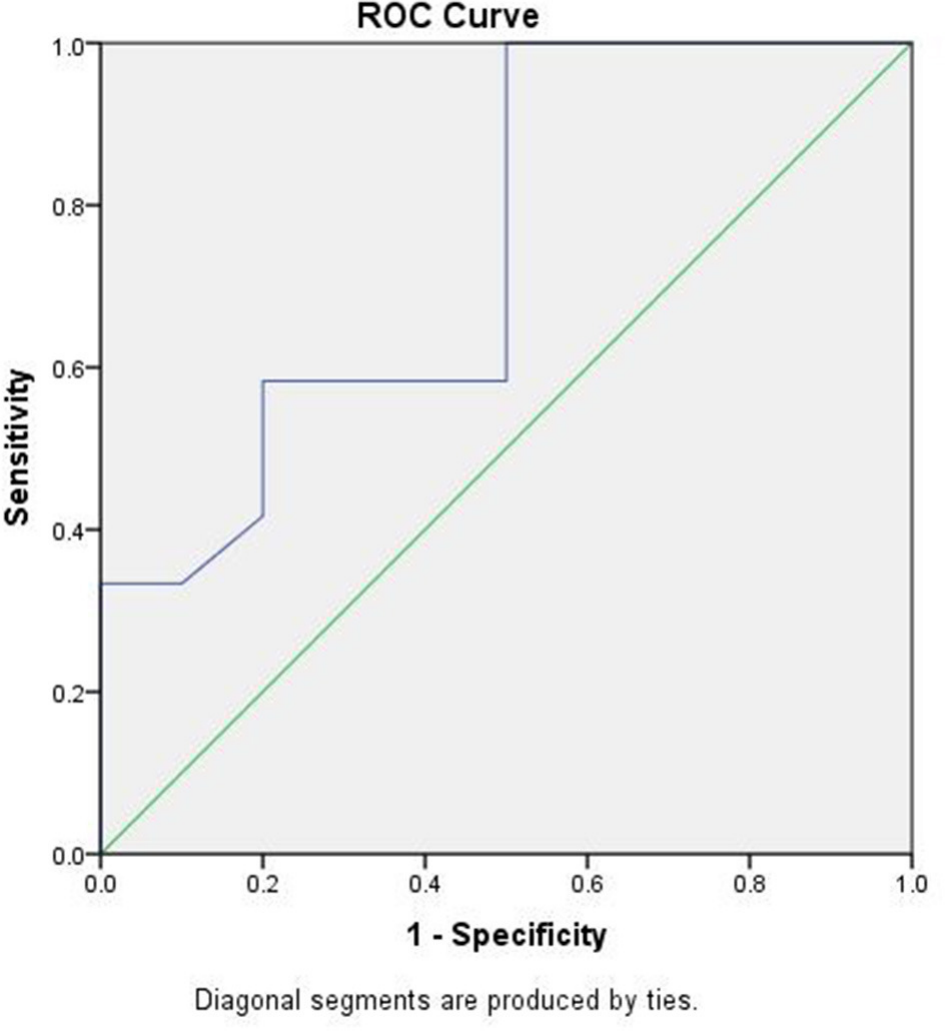
Receiver operater characteristic (ROC) curve of gestational age (GA) at repeat cerclage (RC) predicting delivery ≥28 weeks of gestation. The area under the curve (AUC) is 0.746. GA ≥22.3 weeks at RC had a sensitivity of 100% and specificity of 50%.

Furthermore, the clinical characteristics of singleton pregnancies were compared between GA at delivery of <28 weeks and ≥28 weeks (Table 2). GA at RC was the only risk factor for second-trimester loss. ROC curve analysis demonstrated that GA at RC may be a discriminative parameter for predicting delivery at ≥28 weeks of gestation. The area under curve (AUC) for the prediction of delivery at ≥28 weeks of GA with RC was 0.746 (Figure 1). Using a GA cut-off of 22.3 weeks at RC, the sensitivity and specificity for predicting delivery at ≥28 weeks of gestation were 100% and 50%, respectively.

**Table 2:** Comparison of clinical characteristics among patients with singleton pregnancies between GA at delivery <28 weeks and ≥28 weeks.

	**GA at delivery <28 weeks**	**GA at delivery ≥28 weeks**	***p***
	**(*n* = 10)**	**(*n* = 12)**
Age (years)	34.0 ± 2.5	32.8 ± 3.6	0.124
*In vitro* fertilization (%)	2/10 (20)	1/12 (8.3)	0.571
Gravida	3.7 ± 1.1	2.9 ± 1.0	0.089
Indication of prior cerclage	–	–	
History (%)	6/10 (60)	7/12 (58.3)	1.000
Ultrasound/Physical examination (%)	4/10 (40)	5/12 (41.7)	1.000
Type of suture at prior cerclage	–	–	
5-mm Mersilene® tape (RS 22) (%)	2/10 (20)	4/12 (33.3)	0.646
10^#^ non-absorbable surgical suture (%)	8/10 (80)	8/12 (66.7)	0.646
GA at prior cerclage (weeks)	15.9 ± 1.9	16.4 ± 3.0	0.622
GA at RC (weeks)	22.1 ± 3.1	25.1 ± 2.1	0.015
Cervical dilation (cm)	2.5 ± 0.9	2.1 ± 1.1	0.341
Cervical length (cm)	0.7 ± 1.0	1.2 ± 1.0	0.247
Ureaplasma urealyticum infection (%)	3/10 (30)	3/12 (25)	1.000
Gestational diabetes mellitus (%)	4/10 (40)	4/12 (33.3)	1.000
NLR before RC	5.4 ± 2.5	5.5 ± 3.2	0.953

*Values are presented as n, mean ± standard deviation, or n/N (%)*.

*GA, gestational age; RC, repeat cerclage; NLR, neutrophil-to-lymphocyte ratio*.

**Figure 2:**
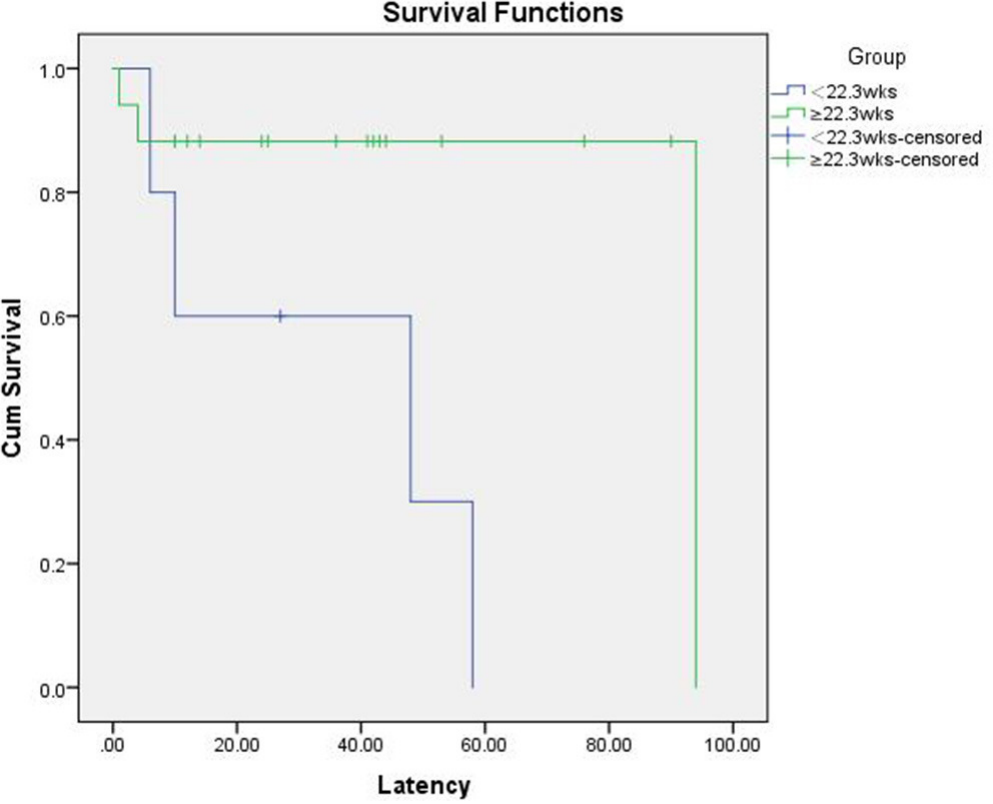
Kaplan–Meier survival curve for neonatal death with gestational age (GA) at repeat cerclage (RC) ≥22.3 weeks and <22.3 weeks.

Based on the cut-off value, patients were divided into a GA <22.3 weeks at RC group and GA ≥22.3 weeks at RC group. Table 3 demonstrates the comparison of pregnancy outcomes between these two groups. The GA <22.3 weeks group showed a worse neonatal outcome compared to the GA ≥22.3 weeks group. There were seven neonatal deaths among all the singleton pregnancies. Kaplan–Meier survival curves showed a lower incidence of neonatal death in the GA ≥22.3 weeks group (17.6%) compared with that in the GA <22.3 weeks group (80%) (p = 0.019, log rank test; Figure 2).

**Table 3:** Comparison of pregnancy outcomes among patients with singleton pregnancies according to GA at RC.

**Variables**	**GA at RC <22.3 weeks (n = 5)**	**GA at RC ≥22.3 weeks (n = 17)**	**p**
GA at delivery (weeks)	23.7 ± 2.9	30.2 ± 3.8	0.002
Latency between RC and delivery (day)	29.8 ± 22.9	36.4 ± 28.8	0.644
Neonatal birth weight (g)	546 ± 326	1372 ± 641	0.012
Live birth rate (%)	1/5 (20)	14/17 (82.4)	0.021
Neonatal survival rate (%)	1/5 (20)	14/17 (82.4)	0.021

*Values are presented as n, mean ± standard deviation, or n/N (%).*.

*GA, gestational age; RC, repeat cerclage*.

## Discussion

To the best of our knowledge, this is the first study investigating the effectiveness of RC for both singleton and twin pregnancies, as well as the first to assess the correlation between clinical factors and pregnancy outcomes following RC in a Chinese population. Evidence from our study suggests that the RC delays delivery by an average of five additional weeks for singleton pregnancies, which is consistent with the findings of Song et al. (15). RC may be an effective method to prolong the duration of pregnancy among patients with singleton pregnancies. On the contrary, RC for twin pregnancies showed an unsatisfactory outcome, such as a poor prolongation of pregnancy and an extremely low neonatal survival rate. This may imply a high risk of delivery before viability after RC for twin pregnancies. The selection of patients with twin pregnancies for RC, thus, remains controversial. Another main finding of our study was that GA at RC appears to be fair for predicting pregnancy outcomes following RC. ROC curve analysis demonstrated that GA at RC could be used to predict delivery at ≥28 weeks of gestation following RC. Our study indicates that the patients who underwent RC at ≥22.3 weeks GA were more likely to deliver after 28 weeks GA, while those who underwent RC at <22.3 weeks of gestation were unlikely to keep the fetus until 28 weeks of gestation. We also found that patients with lower GAs at RC showed worse neonatal outcomes; GA <22.3 weeks at RC was associated with earlier delivery, lower neonatal birth weight, and poorer live birth and neonatal survival rates.

Currently, there are few reports on RC in the literature. Song et al. (15) have suggested that RC improved the pregnancy outcome of patients with prolapsed membranes, including neonatal survival and quality of survival, consistent with our findings. In addition, they also proposed that neutrophil-to-lymphocyte ratio (NLR) might be used as a reliable factor for predicting pregnancy outcomes following RC (17). This was quite different from our finding, and this is likely due to the different populations studied or the limited sample size.

Fox et al. reported that RC appeared to delay delivery by an average of 7 weeks compared to that in the untreated group (16). Althuisius et al. reported the cases of two patients who delivered at term after undergoing RC (18). Baxter et al. reported five cases of ultrasound-indicated reinforcing cerclage; however, the pregnancy outcomes were not improved compared to those in their non-RC counterparts (19). Contag et al. claimed that the placement of RC for short cervix did not prolong the duration of pregnancy, delay GA at delivery, or modify the probability of preterm birth (20). Simcox et al. found that a reinforcing cerclage following primary cerclage failure hastened preterm delivery (21). However, the indications of RC in all the cases reported previously were only identified by ultrasonography after prior cervical cerclage, without the clinical finding of prolapsed membranes.

Predictors of pregnancy outcome following cervical cerclage have become the focus of attention for obstetricians over the last few decades. However, nearly all the studies on predictors of pregnancy outcomes following cervical cerclage have only focused on primary cerclage. Previous studies have shown that pre- and post-cerclage cervical length, positive vaginal culture at cerclage, amniotic fluid levels of neutrophil elastase, and interleukin 6 levels are predictors of latency between cerclage and GA at delivery (3, 5, 23–27). Cervical dilatation, volumetric assessment of cervical funneling, and a history of previous uterine instrumentation are independent predictors of cerclage failure (28–31). GA at emergency cervical cerclage plays an important role on the pregnancy outcome (5, 9, 32). Our study shows, for the first time, that GA at RC is associated with pregnancy outcomes following RC. Furthermore, GA at RC appears to be fair for predicting pregnancy outcomes, and this may help obstetricians identify a better timing to perform RC, predict the effectiveness of RC, and counsel patients with prolapsed amniotic membranes following prior cerclage.

Nevertheless, we acknowledge that there are some limitations to our study. Factors reflecting the severity of cervical insufficiency, such as the GA and indications of the primary cerclage of the patients, may affect the results of the study. However, the heterogeneity of the population and selection bias were not eliminated due to the retrospective design and the limited sample size. In addition, all the patients were of Chinese ethnicity; thus, the conclusions may not be applicable to other populations. Further prospective investigations, including a multicentre, large cohort, are needed in the future.

## Data Availability Statement

The original contributions presented in the study are included in the article/supplementary material, further inquiries can be directed to the corresponding author.

## Ethics Statement

The studies involving human participants were reviewed and approved by Institutional Review Board of Shengjing Hospital of China Medical University. The patients/participants provided their written informed consent to participate in this study.

## Author Contributions

BC designed the study, collected the patients' information, and wrote the manuscript. YX and XN both participated in the collection of the patients' information. All authors approved the final manuscript.

## Funding

This study was supported by the Scientific Research Fund of Liaoning Provincial Education Department (CN) (Award Number: FWZR2020009).

## Conflict of Interest

The authors declare that the research was conducted in the absence of any commercial or financial relationships that could be construed as a potential conflict of interest.

## Publisher's Note

All claims expressed in this article are solely those of the authors and do not necessarily represent those of their affiliated organizations, or those of the publisher, the editors and the reviewers. Any product that may be evaluated in this article, or claim that may be made by its manufacturer, is not guaranteed or endorsed by the publisher.
